# Functional Characterization of Tret1-like Gene and Its Critical Role in Regulating Growth in *Macrobrachium nipponense* by RNA Interference

**DOI:** 10.3390/ijms27177604

**Published:** 2026-08-25

**Authors:** Shubo Jin, Zijian Gao, Yiwei Xiong, Wenyi Zhang, Hongtuo Fu, Hui Qiao, Sufei Jiang

**Affiliations:** 1 Key Laboratory of Freshwater Fisheries and Germplasm Resources Utilization, Ministry of Agriculture and Rural Affairs, Freshwater Fisheries Research Center, Chinese Academy of Fishery Sciences, Wuxi 214081, China; 2 Wuxi Fisheries College, Nanjing Agricultural University, Wuxi 214081, China

**Keywords:** crustacean, qPCR, body mass gain, growth regulation, muscle atrophy

## Abstract

*Macrobrachium nipponense* is a commercially significant freshwater prawn species in China, with larger individuals commanding higher market prices than smaller ones. Previous transcriptomic analyses have shown that a facilitated trehalose transporter gene Tret1-like (*Tret1*) exhibits significantly elevated expression levels in fast-growing individuals compared to their slow-growing counterparts, suggesting a potential involvement of this gene in modulating growth performance. In the present study, we explored the functional roles of *Mn-Tret1* in growth regulation using reverse transcription quantitative real-time PCR (RT-qPCR) and RNA interference (RNAi) techniques. The full-length cDNA sequence of *Mn-Tret1* spans 1605 bp and encodes a 535 amino acid (aa) protein. Phylogenetic analysis indicated that the deduced amino acid sequence of *Mn-Tret1* shares the closest evolutionary relationship with that of *Macrobrachium rosenbergii*. Tissue distribution analysis via RT-qPCR revealed that *Mn-Tret1* is ubiquitously expressed across all examined tissues, implying its potential involvement in multiple physiological processes. Notably, the highest transcript abundance was detected in muscle tissue, further suggesting its putative role in governing growth performance in *M. nipponense.* Subsequent RT-qPCR analysis validated the knockdown efficacy of the synthesized *dsTret1* on *Mn-Tret1* expression. Compared with the *dsGFP*-injected control group, the *dsTret1*-injected group exhibited a statistically significant reduction in body mass gain percentage, with the divergence becoming apparent from day 6 in females and day 12 in males (*p* < 0.05). Moreover, histopathological examination revealed abdominal muscle atrophy in individuals subjected to *dsTret1* treatment. Collectively, these findings substantiate a positive regulatory role of *Mn-Tret1* in governing growth performance. This study thereby contributes to a deeper mechanistic understanding of growth regulation in *M. nipponense*.

## 1. Introduction

*Macrobrachium nipponense* belongs to the phylum Arthropoda, class Malacostraca, order Decapoda, family Palaemonidae, and genus Macrobrachium. According to natural state, it is widely distributed in freshwater and low-salinity estuarine areas of Asian countries such as China, Japan, the Korean Peninsula and Myanmar [1]. In China, it is commonly called Oriental River prawn and it is highly favored by consumers due to its tender texture, delicious taste and high nutritional value, as well as higher contents of trace elements and essential amino acids compared to freshwater species [2]. The output of this prawn exceeded 227,000 tons in 2025, which makes it a representative economic and ecological freshwater aquaculture species in China. The primary aquaculture regions for *M. nipponense* in China include Jiangsu, Anhui, Zhejiang, and Jiangxi provinces, each with an annual production exceeding 15,000 tons [3]. 

Growth performance is a commercially important trait that directly influences the economic value of aquatic animals, as larger individuals generally command a higher market price than smaller ones [4]. Consequently, improving growth traits through genetic selection has long been a primary objective in aquaculture breeding programs. In the recent three decades, three new varieties of *M. nipponense*, named “Taihu No. 1”, “Taihu No. 2”, and “Taihu No. 3”, have been developed through genetic improvement by the Freshwater Fisheries Research Center, Chinese Academy of Fishery Sciences, with body weight as the target trait. These three varieties have been approved as new varieties by the Ministry of Agriculture and Rural Affairs. Notably, the growth rate of “Taihu No. 3” is 30.9% higher than that of the wild *M. nipponense* population from the Yangtze River, making it the main breeding strain of *M. nipponense* in China. 

Growth performance in *M. nipponense* is known to be regulated by multiple factors, including but not limited to stocking density [5,6,7], nutritional status [8,9,10], molting behavior [11], and environmental conditions [12,13]. As a trait of significant economic importance, growth is also heritable, meaning that genetic factors contribute to its phenotypic variation. Consequently, there is an urgent need to identify growth-related genes in *M. nipponense*, which would provide critical evidence for elucidating the molecular mechanisms governing growth in this species. In our previous studies, a combination of genome-wide association study and muscle transcriptomic analysis was applied to screen for potential growth-related genes in *M. nipponense* [14]. From this muscle transcriptomic analysis, two differentially expressed genes (Actin-like and Bestrophin 3) were subsequently validated as being involved in growth regulation in *M. nipponense* [15,16]. Specifically, the expression of both genes was upregulated in fast-growing individuals relative to slow-growing individuals.

From the muscle transcriptomic data described above, a facilitated trehalose transporter Tret1-like gene (*Tret1*) was identified as a candidate growth-related gene in *M. nipponense*, with its expression being upregulated in fast-growing individuals relative to slow-growing ones [14]. Trehalose is a non-reducing disaccharide ubiquitously present across a broad range of organisms, including bacteria, yeasts, fungi, algae, insects, and other invertebrates, where it primarily serves as an energy and carbon source [17]. Owing to its chemical stability, trehalose exhibits strong dehydration resistance and provides effective protection to biomacromolecules under various environmental stresses [18,19]. The trehalose transporters (Tret) are proteins that can transport blood sugar trehalose from the fat body to the hemolymph in insects. They are important transport proteins for maintaining the balance of trehalose in insects and play a significant role in the growth, development, and stress resistance of insects [20,21]. Thus, it is hypothesized that *Tret1* may regulate growth in *M. nipponense* by enhancing trehalose uptake and utilization, thereby supporting energy metabolism in muscle tissue.

In this study, sequence and expression analyses were performed to investigate the genetic information and expression profiles of the *Tret1* gene in *M. nipponense*. Furthermore, RNAi was employed to elucidate its role in growth. The results obtained in this study have direct applications in molecular breeding for improving commercial traits of *M. nipponense*, while simultaneously providing fundamental insights into the regulatory mechanisms governing growth in crustaceans.

## 2. Results

### 2.1. Sequence Information of the Tret1 Gene in M. nipponense

**Figure 1:**
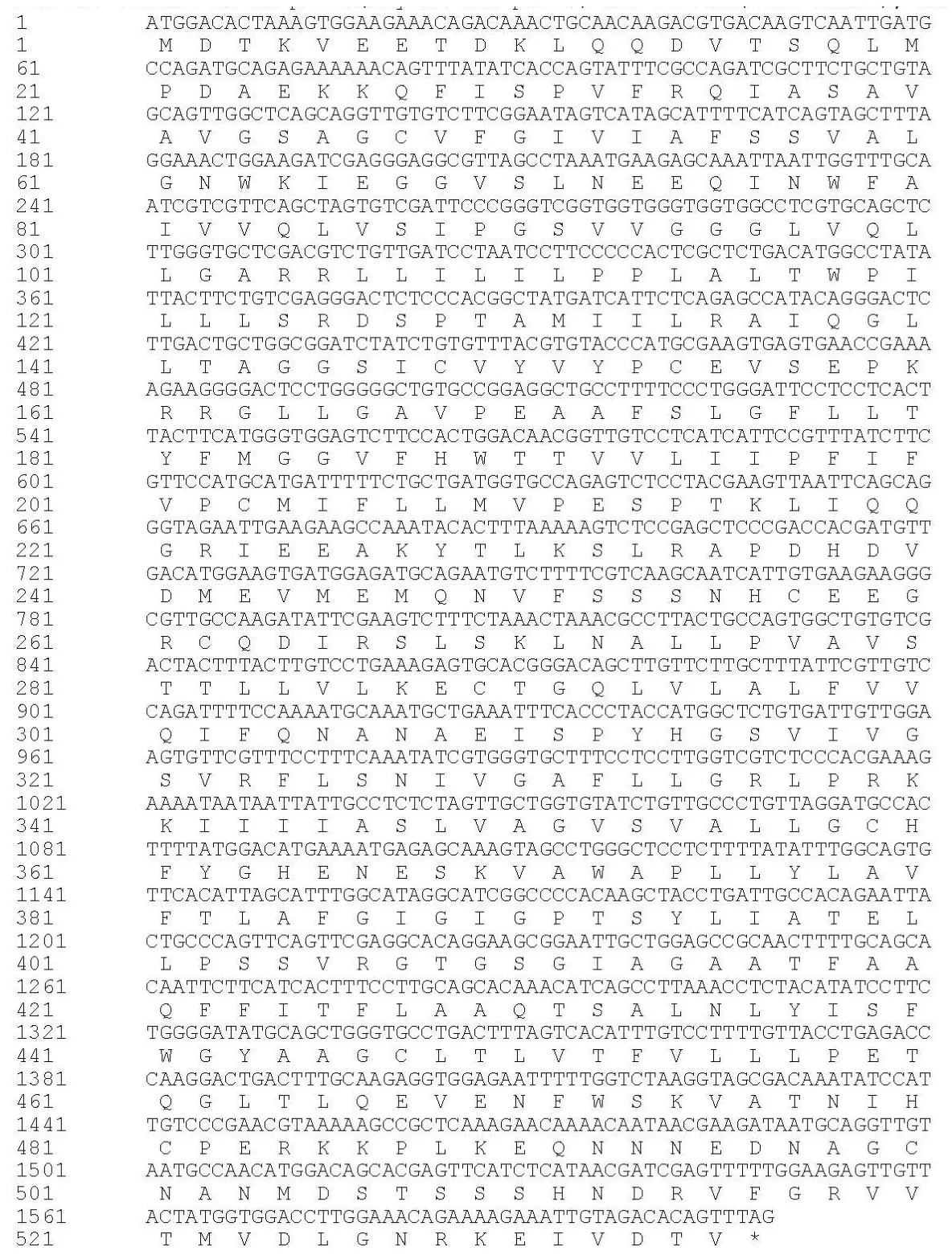
Nucleotide and deduced amino acid sequences of the ORF of *Mn-Tret1*. Both the DNA sequence and the corresponding aa sequence (shown in single-letter uppercase code) are presented in the 5′→3′ orientation. The initiation codon (ATG) and the termination codon (TAA, denoted by an asterisk) are explicitly marked.

**Figure 2:**
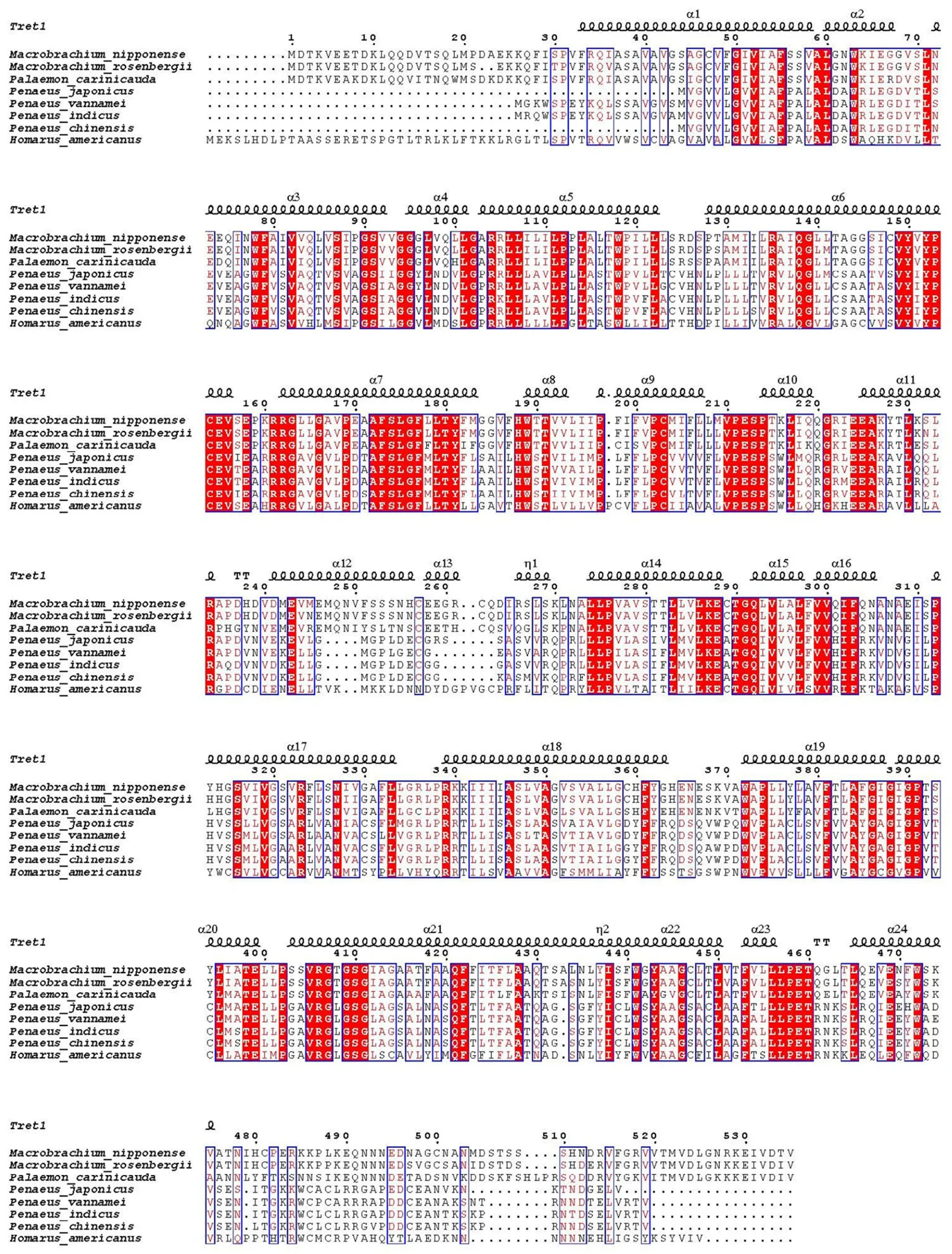
Sequence alignment and structural features of the *Tret1* protein across selected crustacean species. The shading colors denote aa identity levels among species: white letters on a red background in a box for 100%, red letters in the box for ≥50%, and black letters in a box for <50%. A solid black line indicates the conserved major facilitator superfamily (MFS) domain within the *Mn-Tret1* protein. Secondary structure elements are annotated as follows: α, α-helix; TT, β-bend; η, 3_10_-helix.

**Figure 3:**
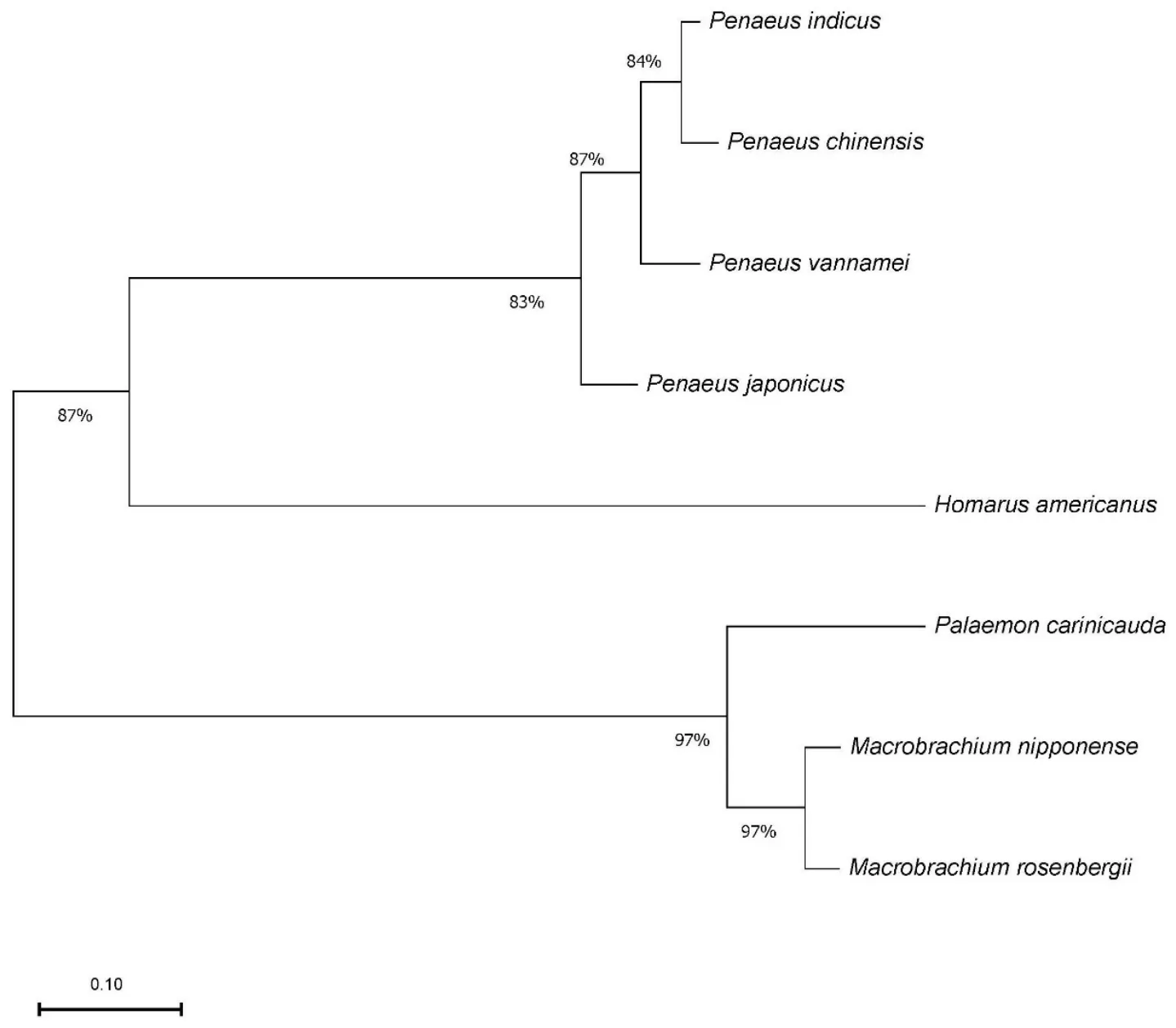
Phylogenetic analysis of the *Tret1* protein among crustacean species.

The open reading frame (ORF) of the *Mn-Tret1* gene is 1605 bp in length, encoding a protein of 535 aa with a predicted molecular mass of 57,856 kDa and an isoelectric point (*pI*) of 5.7051 (Figure 1). The secondary structure of the *Mn-Tret1* protein consists of twenty-four α-helices, one β-turn, and two 3_10_ helices. A conserved functional domain, the major facilitator superfamily (MFS) superfamily domain, was predicted within this protein, spanning aa residues 36 to 469 (Figure 2). Multiple sequence alignment across eight crustacean species revealed that the *Mn-Tret1* gene exhibited relatively low conservation among crustaceans. Only 147 aa sites showed 100% alignment across all species, accounting for approximately 27% of the total aa residues (Figure 2). A phylogenetic tree constructed using the maximum likelihood method based on protein sequences demonstrated that the *Mn-Tret1* shares the highest sequence similarity with that of *M. rosenbergii*, and the two proteins cluster together into the same clade (Figure 3).

### 2.2. Relative Expression of Mn-Tret1 Gene in Different Mature Tissues

**Figure 4:**
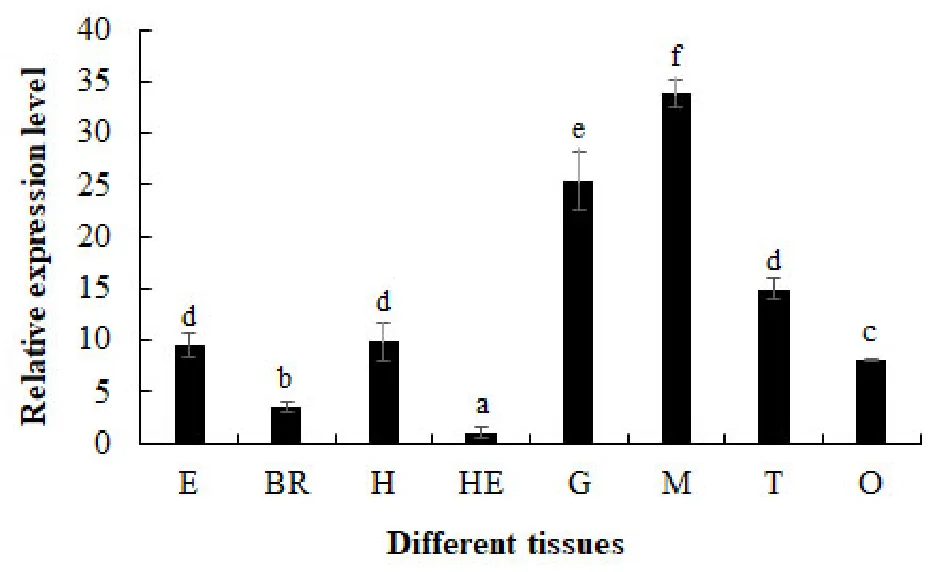
Relative expression levels of *Mn-Tret1* in various mature tissues of *M. nipponense* as determined by RT-qPCR. The *EIF* gene was used as an internal reference for data normalization. Results are presented as mean ± standard deviation (SD, *n* = 6). Distinct lowercase letters above the bars indicate significant differences in *Mn-Tret1* expression among different tissues (*p* < 0.05). Abbreviations: E, eyestalk; BR, brain; H, heart; HE, hepatopancreas; G, gill; M, muscle; O, ovary; and T, testis.

The relative expression levels of *Mn-Tret1* in various tissues were determined by RT-qPCR analysis (Figure 4). The highest expression level of *Mn-Tret1* was observed in muscle tissue, which was significantly higher than that in all other tissues tested (*p* < 0.05). Moderate expression levels were detected in the gills, testis, and heart. In contrast, the lowest expression level was found in the hepatopancreas. Specifically, the expression level of *Mn-Tret1* in muscle tissue was 33.79-fold higher than that in the hepatopancreas.

### 2.3. RNAi Efficiency of dsTret1

**Figure 5:**
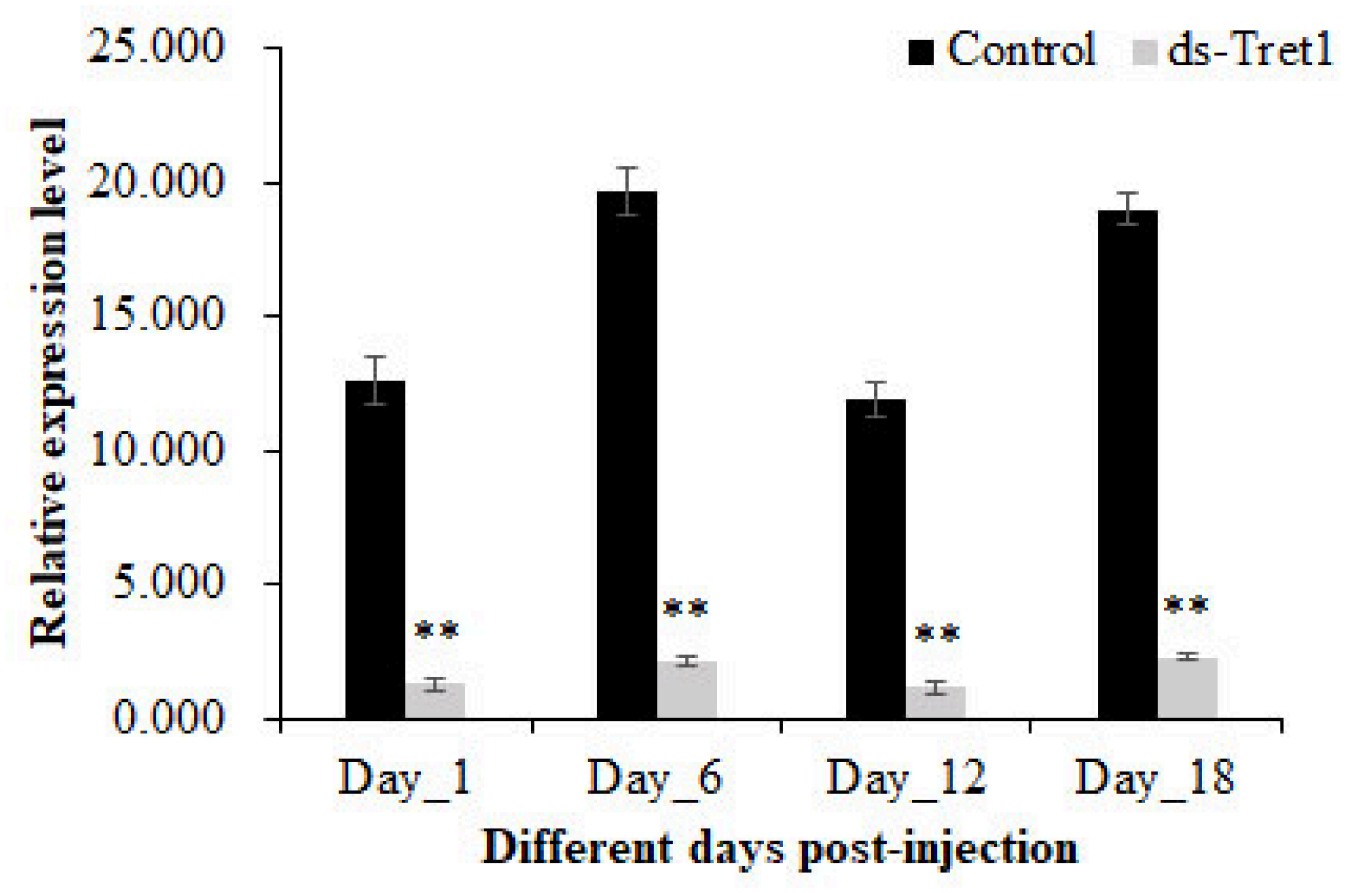
Time-course assessment of *dsTret1*-mediated interference efficiency by RT-qPCR. Relative expression levels of *Mn-Tret1* were normalized to the *EIF* reference gene and are presented as mean ± SD (*n* = 3). Double asterisks (**) denote a highly significant difference between the *dsTret1*-treated group and the *dsGFP*-injected control group at the corresponding time points (*p* < 0.01).

The efficiency of RNAi was assessed by RT-qPCR. The relative expression levels of *Mn-Tret1* were measured on day 1, day 6, day 12, and day 18 after injection of *dsTret1* or *dsGFP*. The results showed that, compared with the control group injected with *dsGFP*, *Mn-Tret1* expression was significantly reduced in the *dsTret1*-treated group at all examined time points (*p* < 0.01). Specifically, *Mn-Tret1* expression was decreased by 89.90%, 89.38%, 90.24%, and 88.09% on day 1, day 6, day 12, and day 18, respectively (Figure 5).

### 2.4. Functional Analysis of Tret1 on Growth of M. nipponense

**Figure 6:**
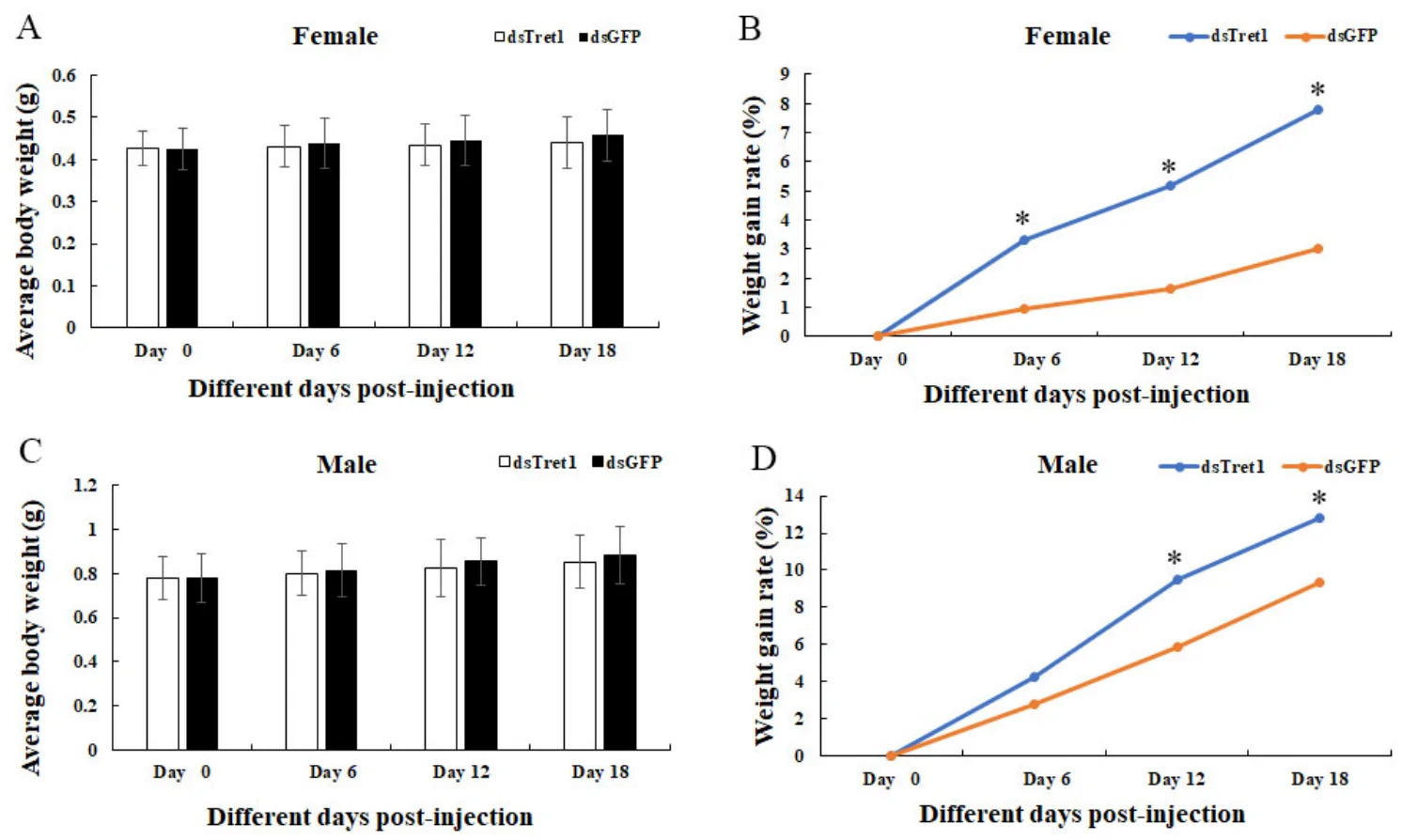
Effects of *dsTret1* injection on body weight gain in *M. nipponense*. Temporal changes in body weight were monitored following *dsTret1* or *dsGFP* (control) injection. At corresponding time points, significant intergroup differences are denoted by asterisks (* *p* < 0.05). (**A**) Body weight gain in females. (**B**) Percentage increase in body mass in females. (**C**) Body weight gain in males. (**D**) Percentage increase in body mass in males.

The initial body weights of female *M. nipponense* in the *dsTret1*-injected group and the *dsGFP*-injected control group were 0.427 ± 0.04 g and 0.424 ± 0.05 g, respectively. In the *dsGFP*-injected control group, body weights increased to 0.438 ± 0.06 g, 0.446 ± 0.06 g, and 0.457 ± 0.06 g on days 6, 12, and 18, respectively. Over the same time points, body weights in the *dsTret1*-injected group reached 0.431 ± 0.05 g, 0.434 ± 0.05 g, and 0.440 ± 0.06 g, respectively (Figure 6A). When the weight gain was calculated as the percentage increase relative to day 0, female prawns in the *dsGFP*-injected control group showed increases of 3.30%, 5.19%, and 7.78% on days 6, 12, and 18, respectively. In comparison, the *dsTret1*-injected group exhibited increases of only 0.938%, 1.64%, and 3.04% at the corresponding time points. A significant difference in the percentage weight increases between the *dsGFP*-injected control group and the *dsTret1*-injected group was observed starting from day 6 post-injection (*p* < 0.05) (Figure 6B).

A similar pattern of weight gain was also observed in male prawns. The initial body weights of male *M. nipponense* were 0.779 ± 0.10 g in the *dsTret1*-injected group and 0.781 ± 0.11 g in the *dsGFP*-injected control group. On days 6, 12, and 18 post-injection, body weights in the *dsGFP*-injected control group increased to 0.814 ± 0.12 g, 0.855 ± 0.11 g, and 0.881 ± 0.13 g, respectively. At the same time points, body weights in the *dsTret1*-injected group reached 0.801 ± 0.10 g, 0.825 ± 0.13 g, and 0.852 ± 0.12 g, respectively (Figure 6C). When expressed as the percentage increase relative to day 0, the *dsGFP*-injected control group showed gains of 4.23%, 9.48%, and 12.80% on days 6, 12, and 18, respectively. Over the same period, the *dsTret1*-injected group exhibited corresponding increases of 2.82%, 5.91%, and 9.37%. A statistically significant difference in the percentage weight gain between the *dsGFP*-injected control and *dsTret1*-injected groups was detected starting from day 12 post-injection (*p* < 0.05) (Figure 6D).

At the conclusion of the experiment (18 days post-injection), all surviving prawns were counted, and their average daily weight gain (ADWG) was calculated. In the *dsTret1*-injected group, the ADWG for females was 1.693 ± 1.092‰, compared to 4.239 ± 1.831‰ in the *dsGFP*-injected control group. Similarly, for males, the ADWG in the *dsTret1*-injected group was 5.122 ± 1.229‰, whereas it was 6.993 ± 0.563‰ in the *dsGFP*-injected control group. For both sexes, the ADWG values in the *dsTret1*-injected group differed significantly from those in the *dsGFP*-injected control group (*p* < 0.05) (Table 1).

### 2.5. Morphological Difference in the Muscle Tissue Between dsTret1-Injected and dsGFP-Injected Group

**Figure 7:**
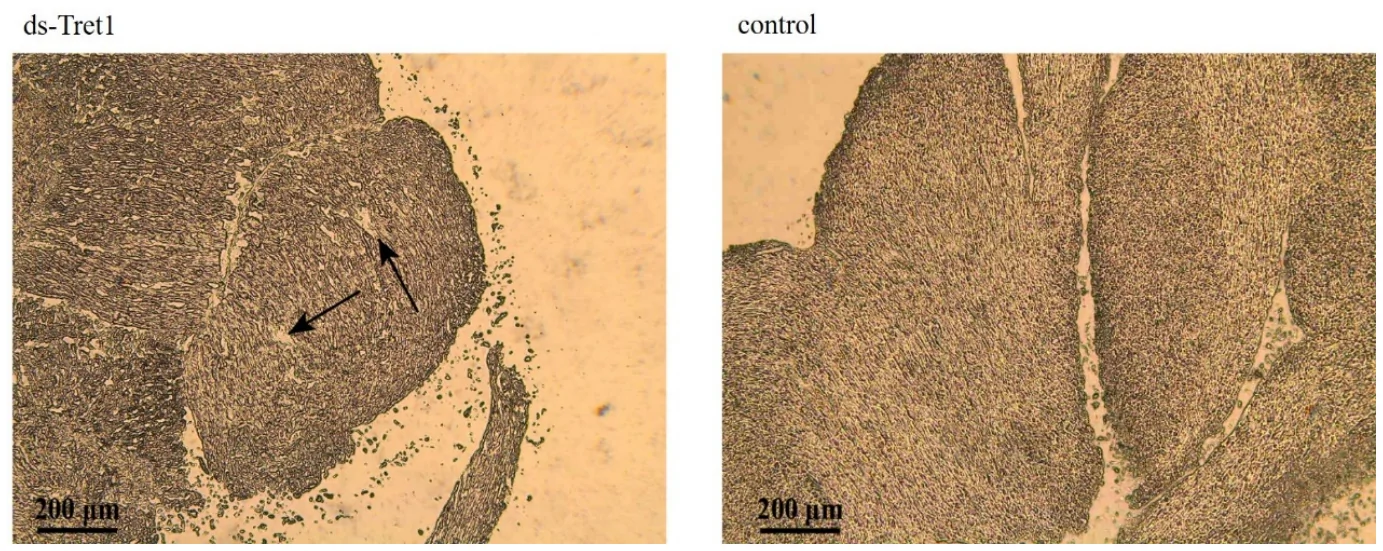
Histological examination of abdominal muscle tissue at 18 days post-injection with *dsTret1* or *dsGFP* (control). The arrows indicate regions of myofibrillar erosion and disorganization observed in the *dsTret1*-treated group.

To compare muscle tissue morphology between the *dsTret1*-injected and *dsGFP*-injected groups, abdominal muscle samples were harvested at 18 days post-injection and examined via frozen sectioning. Compared with the *dsGFP*-injected control group, the *dsTret1*-injected prawns displayed distinct erosive changes in their myofibrillar tissues, which consequently led to an enlargement of the intermyofibrillar spaces. In the dsGFP-injected group, the muscle bundle diameter and intermyofibrillar space were 7.91 ± 1.41 µm and 5.68 ± 0.95 µm, respectively, whereas in the dsTret1-injected group, these values were 5.35 ± 1.14 µm and 8.58 ± 0.61 µm (*p* < 0.05). These morphological alterations suggested the occurrence of abdominal muscle atrophy in the *dsTret1*-treated individuals (Figure 7).

## 3. Discussion

In insects, *Tret* functions as a high-capacity carrier that directly mediates the transport of newly synthesized trehalose from the fat body across the cell membrane into the extracellular space (hemolymph), from which it is distributed to various tissues to meet the energy demands of physiological activities [20,21]. Beyond its role in energy supply, *Tret1* also plays important roles in insect physiological metabolism and stress resistance [22]. In *Hyphantria cunea*, *Tret1* has been shown to play an essential role in both the larva–pupa transition and the maintenance of systemic trehalose homeostasis, with particular involvement in epidermal chitin biosynthesis during the larval stage [23]. In *Anopheles gambiae*, *Tret1* not only mediates trehalose transport but also contributes significantly to the mosquito’s physiological resilience against environmental stressors and its responsiveness to malaria parasite infection [17]. Previous investigations from our laboratory demonstrated that *Tret1* expression was upregulated in fast-growing *M. nipponense* relative to slow-growing individuals, indicating that this gene may contribute to growth regulation in this prawn species [14]. On the basis of this observation, the present study was undertaken to investigate the role of *Tret1* in governing growth performance in *M. nipponense*.

*Tret1* transcripts were investigated in insects, including the stages during larval development [24] and treatment after environmental stress [25,26]. In aquatic animals, transcriptome analysis coupled with RT-qPCR validation revealed that *Tret1* transcript levels in the ovary of *Penaeus vannamei* were higher than those in the testis [27]. Additionally, transcriptome profiling demonstrated that *Tret1* expression in the hepatopancreas was significantly downregulated under low-salinity stress, indicating that this gene is responsive to environmental salinity changes [28]. However, to the best of our knowledge, no study has yet employed RT-qPCR to systematically examine *Tret1* expression across all mature tissues in any aquatic species. In this study, *Mn-Tret1* transcripts were ubiquitously distributed across all examined tissues, implying a pleiotropic role for this gene in *M. nipponense*. Among these, the most abundant expression was consistently found in muscle tissue. It is well established that muscle-enriched genes can shape growth phenotypes in aquatic organisms via diverse molecular cascades. For example, the insulin-like growth factor axis (*IGF-1* and *IGF-2*), along with its receptor system and binding proteins, is actively transcribed in muscle, where it coordinates anabolic effects by upregulating protein synthesis and attenuating proteolysis, thus, driving both myofiber hypertrophy and hyperplasia [29,30]. Concurrently, the myogenic regulatory factor family exerts hierarchical control over the commitment, expansion, and terminal fusion of muscle progenitor cells into mature myofibers [31,32]. The transcriptional activity of these regulators correlates tightly with myofiber size and abundance, which in turn dictate ultimate body dimensions and somatic growth rates. Notably, prior investigations have identified two other muscle-predominant genes (actin-like gene and bestrophin 3) as positive modulators of growth in this species [15,16]. Accordingly, our spatial expression profiling points to a potential involvement of *Mn-Tret1* in growth regulation, a finding that resonates well with earlier observations.

RNAi constitutes an evolutionarily conserved mechanism that functions both in cellular defense and in the regulation of endogenous gene expression. This technique employs small non-coding RNA molecules as sequence-specific guides to recognize and cleave complementary messenger RNA targets, thereby achieving post-transcriptional gene silencing [33]. To date, RNAi has been documented in a wide range of eukaryotic organisms, including crustaceans, in which it participates in multiple biological processes such as antiviral host defense, repression of transposable elements, orchestration of developmental programs, and acclimation to environmental changes [34,35,36]. RNAi-mediated knockdown of *Tret1* in *Trichogramma dendrolimi* led to a delayed larval–prepupal transition during the larval developmental stage, as well as disrupted ovarian development during the adult stage [24]. In *H. cunea*, RNAi-mediated knockdown of *HcTRET1* did not affect larval growth, development, or survival rate; however, it significantly reduced the pupation rate. Moreover, silencing of *HcTRET1* led to elevated trehalose levels in the body fat but decreased levels in the hemolymph, indicating that *HcTRET1* plays a critical role in maintaining trehalose homeostasis [23]. In *Plutella xylostella*, RNAi-mediated knockdown of *Tret1* expression severely impaired adult fitness, manifesting as shortened lifespan, wing deformities, and reduced fecundity [25]. In *Psylliodes chrysocephala*, RNAi-based assays demonstrated that *Tret1* functions in the transport of trehalose from the fat body to the hemolymph [19]. However, to date, the potential function of this gene in regulating growth performance has not been investigated in any species. Moreover, to the best of our knowledge, studies on the functional roles of *Tret1* in aquatic animals are limited. The RNAi technique has been well established in *M. nipponense* [37], and its utility has been demonstrated through functional characterization of numerous genes via RNAi-based approaches in this species [38,39,40,41,42,43]. In the present study, injection of *dsTret1* resulted in an 88.09–90.24% reduction in *Mn-Tret1* transcript levels relative to the *dsGFP*-injected control group at corresponding time points, confirming that the synthesized *dsTret1* effectively silences *Mn-Tret1* expression in this species. In both males and females, *dsTret1* injection significantly impaired growth performance, as evidenced by a marked suppression of body weight gain beginning on day 6 post-injection in females and on day 12 in males. Moreover, histological examination of muscle tissue from *dsTret1*-treated prawns revealed an enlargement of intermyofibrillar spaces, indicative of abdominal muscle atrophy. Collectively, these results demonstrated that *Tret1* positively regulated growth performance in *M. nipponense*. A possible explanation is that *Tret1* knockdown disrupts energy metabolism, thereby compromising growth. Nevertheless, additional studies are needed to substantiate this hypothesis. Interestingly, a stronger regulatory effect of this gene on growth was observed in female shrimp than in male shrimp. This disparity is possibly explained by the inherently higher growth rate and metabolic rate in males, which may result in differential responsiveness to the modulation of growth by this gene.

## 4. Materials and Methods

### 4.1. cDNA Sequences Characterization of Mn-Tret1

The cDNA sequences of *Tret1* gene in *M. nipponense* were obtained from the whole-genome database (GenBank accession number: GCA_015104395.2) and muscle transcriptome database (GenBank accession numbers: SRX25177010–SRX25177021) of *M. nipponense*. From 10 individual prawns, 0.1 g of muscle tissue was collected per specimen, and the RNAiso Plus Reagent kit (TaKaRa, Dalian, China) was subsequently applied for total RNA extraction from each sample. The concentration of extracted RNA was measured using a spectrophotometer (Eppendorf, Hamburg, Germany), whereas its integrity was examined by agarose gel electrophoresis. For each sample, approximately 1 µg of total RNA was used to synthesize first-strand cDNA through reverse transcription employing the iScript™ cDNA Synthesis Kit (Bio-Rad, Hercules, CA, USA). To verify the accuracy of the obtained coding-region sequence, experimental validation was carried out via PCR amplification with a pair of primers, using the synthesized cDNA as the template. The primers designed for coding-region sequence verification were generated using the Primer-BLAST online tool and are presented in Table 2 (https://www.ncbi.nlm.nih.gov/tools/primer-blast/, accessed on 12 June 2024). A 25 µL PCR reaction system was prepared, consisting of 0.5 µL of forward primer (10 µM), 0.5 µL of reverse primer (10 µM), 1 µL of cDNA template, 19.9 µL of DEPC-treated water, 2.5 µL of 10× PCR supermix, 0.5 µL of dNTP mix (10 mM), and 0.1 µL of Taq DNA polymerase. The thermocycling conditions were set as follows: initial denaturation at 95 °C for 3 min; 35 cycles of 95 °C for 30 s, 62 °C for 60 s, and 72 °C for 30 s; followed by a final extension at 72 °C for 10 min. The amplified PCR product was then cloned into the PMD-18T vector (Takara, Dalian, China), and DNA sequencing was conducted on an ABI 3730 automated sequencer (Invitrogen Biotechnology Co., Ltd., Carlsbad, CA, USA). Ultimately, the PCR products were submitted to Sangon Biotech (Shanghai, China) for sequencing.

### 4.2. Sequence Structure Analysis of Mn-Tret1 Gene

The ORFs of *Mn-Tret1* were predicted using the ORF-FINDER web tool (https://www.ncbi.nlm.nih.gov/orffinder/, accessed on 19 June 2024) [44]. The *pI* and molecular weight of the *Mn-Tret1* protein were calculated using the Compute pI/Mw tool available at the ExPASy server (https://web.expasy.org/compute_pi/, accessed on 19 June 2024). The protein sequences encoded by the target gene cDNA were converted and visualized using DNAman (version 6.0) software. The secondary structure domains were predicted using the SWISS-PROT database [45], and the homologous domains were predicted using the CDD database [46].

The *Tret1* protein sequences for other crustaceans, such as *M. rosenbergii* (XP_066972120.1), *P. vannamei* (XP_027236048.2), *Penaeus chinensis* (XP_047502990.1), *Penaeus japonicus* (XP_042877816.1), *Homarus americanus* (XP_042217504.1), *Palaemon carinicauda* (XP_068233900.1), and *Penaeus indicus* (XP_063605138.1) were downloaded from the GenBank database [47]. The protein sequences then were compared using the ClustalW (version 2.0) [48]. Based on the results, the phylogenetic tree was constructed using MEGA (version 11) [49] based on 1000 repeated sampling (bootstrap = 1000). The overall support rate was displayed at the nodes of the phylogenetic tree.

### 4.3. Expression Profiles Analysis of Tret1 Gene

Total RNA was isolated from a range of adult prawn tissues, including eyestalks, brain ganglia, heart, hepatopancreas, gills, muscle, testes, and ovaries. For the testes and ovaries, a single biological replicate was generated by combining tissue samples from three individuals of the same sex. For the remaining tissues, samples were collected separately from three males and three females, then pooled to form one biological replicate. Six of such biological replicates were prepared for each tissue type. The detailed procedures for RNA extraction and cDNA synthesis are described in Section 2.1. The RT-qPCR reaction and thermal cycling conditions followed those described in a previous study [50]. The eukaryotic translation initiation factor 5A gene (*EIF*) was used as an internal reference gene [51]. Primers for RT-qPCR analysis were also designed using the Primer-BLAST online tool (https://www.ncbi.nlm.nih.gov/tools/primer-blast/, accessed on 20 June 2024). The primer sequences for both *EIF* and *Mn-Tret1* are listed in Table 2. The amplification efficiencies of *Mn-Tret1* and *EIF* were confirmed to be approximately comparable. Consequently, the relative expression levels of *Mn-Tret1* were calculated using the 2^−ΔΔCT^ method [52].

### 4.4. RNAi Analysis of Mn-Tret1 Gene

To investigate the potential role of *Tret1* in regulating growth performance in *M. nipponense*, RNAi was employed in this study. The green fluorescent protein (*GFP*) gene was utilized as a negative control [53]. Double-stranded RNA (*dsRNA*) primers of *Mn-Tret1* were designed using the SnapDragon web tool (https://www.flyrnai.org/cgi-bin/RNAi_find_primers.pl, accessed on 12 July 2024), with a T7 promoter sequence (5′-TAATACGACTCACTATAGGG-3′) appended to the 5′ terminus (Table 2). The *dsRNA* for both target and control genes was synthesized using a TranscriptAid™ T7 High-Output Transcription Kit (Fermentas, Waltham, MA, USA) according to the manufacturer’s recommended protocol. From the same *M. nipponense* population, 200 individuals (100 males and 100 females) and 240 individuals (120 males and 120 females) were collected, and assigned to the RNAi efficiency assay and the growth performance assay, respectively.

RNAi efficiency assay: A total of 100 female (0.417 ± 0.04 g) and 100 male (0.812 ± 0.10 g) prawns were obtained and randomly allocated to either a *dsGFP*-injected control group or a *dsTret1*-injected experimental group, with each group comprising 50 individuals of each sex. Twenty-five single-sex *M. nipponense* were reared in each tank with a volume of 1 m^3^ of water. Prior to and during the injection period, all animals were acclimatized under laboratory conditions (water temperature: 27 ± 1 °C; pH: 7.37–7.64; dissolved oxygen: >6.0 mg/L; photoperiod: 14–16 h) and fed twice daily with fresh snail meat at a rate of 3% of total body weight. According to a previously established protocol [36], prawns in the experimental group received microinjections of *dsTret1* via the pericardial cavity, while those in the control cohort were injected with *dsGFP*. The *dsRNA* solutions were prepared at a concentration of 4 µg/µL in isotonic vehicle, and each animal was administered a dose of 4 µg per gram of body weight. Under this dosing regimen, the injection volume (in µL) for each prawn was numerically equivalent to its body weight (in grams). Both groups received injections of *dsTret1* or *dsGFP* once every 6 days, for a total of three injections. To evaluate the knockdown efficiency, *Mn-Tret1* transcript levels in muscle tissue were quantified by RT-qPCR at four post-injection time points: day 1, day 6, day 12, and day 18. At each time point, muscle tissues were collected from four individuals (two males and two females) and pooled to constitute one biological replicate; three such replicates were prepared per time point. The procedures for RNA extraction, cDNA synthesis, and qPCR analysis were performed as detailed in Section 4.1 and Section 4.3.

Growth performance assay: A total of 120 male prawns (initial body weight: 0.75–0.82 g) and 120 female prawns (initial body weight: 0.41–0.45 g) were randomly allocated into two experimental groups: a *dsGFP*-injected control group and a *dsTret1*-injected experimental group, each comprising 60 male or 60 female individuals. The mean initial body weights (±SD) were 0.781 ± 0.11 g for males and 0.424 ± 0.05 g for females in the control group, and 0.779 ± 0.10 g for males and 0.427 ± 0.04 g for females in the *dsTret1*-treated group. Twenty single-sex *M. nipponense* were reared in each tank with a volume of 1 m^3^ of water. Thus, individuals of both sexes from the *dsGFP*-injected control group and the *dsTret1*-injected experimental group were randomly assigned to three independent tanks, representing three biological replicates. Prior to and during the injection period, all experimental prawns were maintained under the same rearing and feeding conditions as those described previously. The synthesis and injection methods for *dsRNA* were identical to those described above. Body weight measurements were recorded for each prawn on the same days as the injections. Weight gain rate (WGR) was calculated as follows: WGR = (W_n+1_ − W_n_)/W_n_ × 100%. The Shapiro–Wilk test and Levene’s test were applied to assess the normality of distribution and the homogeneity of variances between the two groups, respectively. Based on these results, either Student’s *t*-test (for equal variances) or Welch’s *t*-test (for unequal variances) was used to compare group differences. At the end of the experiment (18 days post-injection), all surviving prawns were counted, and the ADWG was calculated as: ADWG = (final weight − initial weight)/feeding days.

### 4.5. Histological Observations of Muscle Tissue

At 18 days, samples were collected from the widest portion of abdominal muscle, and a histological analysis was carried out to compare tissue morphology between the two groups. Muscle tissues from each group were embedded using an optimal cutting temperature (OCT) embedding kit (Rebiosci, Shanghai, China), following the manufacturer’s instructions. Longitudinal sections were then obtained using an OTF5000 cryostat (Bright, San Francisco, CA, USA). For histological assessment, muscle tissue samples were cut into 10 µm thick cryosections. The resulting sections were examined and observed at 400× magnification under a light microscope (LEICA MC170 HD, Wetzlar, Germany).

### 4.6. Statistical Analysis

SPSS Statistics 23.0 was employed for all statistical analyses. One-way ANOVA was used to determine significant differences among groups, with LSD and Duncan’s post hoc tests applied for multiple comparisons. Before conducting the ANOVA, the Shapiro–Wilk test was used to assess data normality, and Levene’s test was performed to check for homogeneity of variances. The quantitative data are expressed as the mean ± standard deviation (SD), and statistical significance was defined as a *p*-value less than 0.05.

## 5. Conclusions

The full-length cDNA of *Mn-Tret1* comprises 1605 bp and encodes a protein of 535 aa. Phylogenetic analysis based on the deduced aa sequence indicates that *Mn-Tret1* is evolutionarily most closely related to its ortholog in *M. rosenbergii*. Tissue distribution profiling revealed that *Mn-Tret1* transcript abundance is highest in muscle tissue. Furthermore, following RNAi-mediated knockdown, the *dsTret1*-injected group showed a significantly lower percentage increase in body mass relative to the *dsGFP*-injected controls (*p* < 0.05). Histological examination also revealed pronounced atrophy of the abdominal musculature in *dsTret1*-treated individuals. Taken together, these results establish a positive regulatory function of *Mn-Tret1* in modulating growth performance. In future studies, single nucleotide polymorphisms (SNPs) associated with growth traits should be identified within this gene to facilitate the genetic improvement of growth performance in this species via molecular-assisted selection. In addition, the molecular mechanism underlying the growth-regulatory function of this gene will be further investigated by measuring changes in trehalose and hemolymph sugar levels following RNAi-mediated knockdown of *Tret1* expression.

## Figures and Tables

**Table 1:** Average ADWG after 18 days post-injection.

Gender	Group	ADWG (‰)
Female	*Tret1-like*	1.693 ± 1.092 *
	Control	4.293 ± 1.831
Male	*Tret1-like*	5.122 ± 1.229 *
	Control	6.993 ± 0.563

Asterisks (*) indicate a significant difference between *dsTret1* and *dsGFP* injection (*p* < 0.05).

**Table 2:** Primers used in this study.

Primer	Primer Sequence (5′-3′)	Purpose
F1	TTGGTGCGACGCAGAAGGGAGT	ORF verification
R2	TTTCTGTTTCCAAGGTCCACCA	
qPCR-F	CTTTCCTTGCAGCACAAACA	qPCR analysis
qPCR-R	GTGTGATTGGTAATTGCACTCGT	
*EIF*-F	CATGGATGTACCTGTGGTGAAAC	
*EIF*-R	CGTGCACTCTTTCAGGACAA	
RNAi-F	TAATACGACTCACTATAGGGCACTTTCCTTGCAGCACAAA	RNAi analysis
RNAi-R	TAATACGACTCACTATAGGGTGTTCTTTGAGCGGCTTTTT	
*GFP*-F	TAATACGACTCACTATAGGGGTCAGTGGAGAGGGTGAAGG	
*GFP*-R	TAATACGACTCACTATAGGGGAAAGGGCAGATTGTGTGGAC	

## Data Availability

The original contributions presented in this study are included in the article. Further inquiries can be directed to the corresponding authors.

## References

[1] Wu P., Qi D., Chen L., Zhang H., Zhang X., Qin J.G., Hu S. (2009). Gene discovery from an ovary cDNA library of oriental river prawn *Macrobrachium nipponense* by ESTs annotation. Comp. Biochem. Physiol. Part D Genom. Proteom..

[2] Kong Y., Ding Z., Zhang Y., Zhou P., Wu C., Zhu M., Ye J. (2019). Types of carbohydrate in feed affect the growth performance, antioxidant capacity, immunity, and activity of digestive and carbohydrate metabolism enzymes in juvenile *Macrobrachium nipponense*. Aquaculture.

[3] China Agricultural Press (2026). Fisheries Economic Statistics. China Fishery Yearbook.

[4] Tsikliras A.C., Stergiou K.I. (2014). Fish market prices drive overfishing of the ‘big ones’. PeerJ.

[5] Sun S.M., Fu H.T., Gu Z.M., Zhu J. (2016). Effects of stocking density on the individual growth and differentiation of the oriental river prawn *Macrobrachium nipponense* (de haan, 1849) (caridea: Palaemonidae). J. Crustac. Biol..

[6] Ettefaghdoost M., Babakhani A. (2025). Effects of stocking density on the oriental river prawn (*Macrobrachium nipponense*): A comprehensive evaluation of growth, hematological, physio-immunological, and antioxidant responses. Aquacult. Int..

[7] Xu M., Xiong Y.W., Xiao Z.Q., Zhang W.Y., Fu H.T., Qiao H., Jiang S.F. (2026). Stocking density effects on growth performance, water quality, immune indexes, and economic benefits of oriental river prawns (*Macrobrachium nipponense*) reared in earthen ponds. Aquacult. Rep..

[8] Dong Y.Z., Wei L.Y., Liu W.B., Liu B., Sun C.X., Li X.F. (2026). Optimal dietary pyridoxine requirement of oriental river prawn (*Macrobrachium nipponense*): Insights from growth, feed utilization, and protein metabolism. Anim. Feed Sci. Technol..

[9] Ettefaghdoost M., Haghighi H., Babakhani A. (2026). Curcumin as a functional feed additive enhances growth, immunity, antioxidant defense, gut microbiota, muscle quality, and disease resistance in the oriental river prawn (*Macrobrachium nipponense*). Appl. Food Res..

[10] Ettefaghdoost M., Haghdoost A. (2026). Functional role of dietary bixin as a natural carotenoid: Integrative modulation of growth, metabolism, antioxidant defense, and disease resistance in the oriental river prawn (*Macrobrachium nipponense*). Aquaculture.

[11] Wang W.N., Wang A.L., Nu D.H., Liu Y.C., Sun R.Y. (2005). Effects of molting on the growth of primary cultured muscle cells from *Macrobrachium nipponense* and *Penaeus vannamei*. Isr. J. Aquac.-Bamidgeh.

[12] Ding Z.L., Kong Y.Q., Shao X.P., Zhang Y.X., Ren C.C., Zhao X.M., Yu W.S., Jiang T.Q., Ye J.Y. (2019). Growth, antioxidant capacity, intestinal morphology, and metabolomic responses of juvenile Oriental river prawn (*Macrobrachium nipponense*) to chronic lead exposure. Chemosphere.

[13] Li Y.M., Du X.L., Jiang Q.C., Huang Y.Y., Zhao Y.L. (2022). Effects of nanoplastic exposure on the growth performance and molecular characterization of growth-associated genes in juvenile *Macrobrachium nipponense*. Comp. Biochem. Physiol. Part C Toxicol. Pharmacol..

[14] Gao Z., Zhang W., Jiang S., Qiao H., Xiong Y., Jin S., Fu H. (2024). Genome-wide association and transcriptomic analysis and the identification of growth-related genes in *Macrobrachium nipponense*. BMC Genom..

[15] Jin S.B., Lin J.Y., Fu H.T., Xiong Y.W., Qiao H., Zhang W.Y., Jiang S.F. (2026). RNAi Identified the potential functions of actin-like protein in the growth performance of *Macrobrachium nipponense*. Int. J. Mol. Sci..

[16] Jin S.B., Gao Z.J., Fu H.T., Xiong Y.W., Qiao H., Zhang W.Y., Jiang S.F. (2026). Identification of potential roles of bestrophin 3 in the growth performance of Oriental river prawn *Macrobrachium nipponense* by RNA interference. Int. J. Mol. Sci..

[17] Liu K., Dong Y.M., Huang Y.Z., Rasgon J.L., Agre P. (2013). Impact of trehalose transporter knockdown on *Anopheles gambiae* stress adaptation and susceptibility to *Plasmodium falciparum* infection. Proc. Natl. Acad. Sci. USA.

[18] Lu K., Wang Y., Chen X., Zhang X., Li W., Cheng Y., Li Y., Zhou J., You K., Song Y. (2019). Adipokinetic hormone receptor mediates trehalose homeostasis to promote vitellogenin uptake by oocytes in *Nilaparvata lugens*. Front. Physiol..

[19] Güney G., Cedden D., Scholten S., Rostás M. (2025). Reciprocal roles of two trehalose transporters in aestivating cabbage stem flea beetle (*Psylliodes chrysocephala*). Insect Biochem. Mol. Biol..

[20] Kikawada T., Saito A., Kanamori Y., Nakahara Y., Iwata K., Tanaka D., Watanabe M., Okuda T. (2007). Trehalose transporter 1, a facilitated and highcapacity trehalose transporter, allows exogenous trehalose uptake into cells. Proc. Natl. Acad. Sci. USA.

[21] Kanamori Y., Saito A., Hagiwara-Komoda Y., Tanaka D., Mitsumasu K., Kikuta S., Watanabe M., Cornette R., Kikawada T., Okuda T. (2010). The trehalose transporter 1 gene sequence is conserved in insects and encodes proteins with different kinetic properties involved in trehalose import into peripheral tissues. Insect Biochem. Mol. Biol..

[22] Li J.X., Cao Z., Guo S., Tian Z., Liu W., Zhu F., Wang X.P. (2020). Molecular characterization and functional analysis of two trehalose transporter genes in the cabbage beetle, *Colaphellus bowringi*. J. Asia-Pac. Entomol..

[23] Liu D., Zou C., Zhang S., Wang Z., Yu J., Nan Y., Dong Z. (2025). HcTRET1 is critical for epidermal chitin synthesis in *Hyphantria cunea*. Insect Mol. Biol..

[24] Zhang X., Song X.Y., Zheng M.X., Guo J.Q., Fan Y.N., Huang J.B., Zhang Q.Y., Zhang J.J., Ruan C.C. (2026). Dual-function trehalose transporters link metabolic adaptation to diapause plasticity and fecundity in *Trichogramma dendrolimi* (Hymenoptera: Trichogrammatidae). J. Insect Physiol..

[25] Wang Y.P., Fu Q.R., Xie L.Y., Wei P.P., Refukaiti A., Xia B.L., Song Q.S., Xie M. (2026). PxTret1-mediated trehalose transport regulates development and reproduction in *Plutella xylostella* with implications for Cry1Ac tolerance. Pestic. Biochem. Physiol..

[26] Zou Y., Xiong W.H., Liao B.B., Kou Y.N., Xin T.R., Wan B., Xia B., Zou Z.W. (2024). Continuous improvement of cold tolerance in *Aleuroglyphus ovatus* is achieved through the enhancement of trehalose transport facilitated by genes encoding trehalose transporters. J. Stored Prod. Res..

[27] Peng J.X., Wei P.Y., Zhang B., Zhao Y.Z., Zeng D.G., Chen X.L., Li M., Chen X.H. (2015). Gonadal transcriptomic analysis and differentially expressed genes in the testis and ovary of the Pacific white shrimp (*Litopenaeus vannamei*). BMC Genom..

[28] Cao S., Li Y., Jiang S., Yang Q., Huang J., Yang L., Shi J., Jiang S., Wen G., Zhou F. (2024). Transcriptome Analysis Reveals the Regulatory Mechanism of Lipid Metabolism and Oxidative Stress in *Litopenaeus vannamei* under Low-Salinity Stress. J. Mar. Sci. Eng..

[29] Blum W.F., Alherbish A., Alsagheir A., El Awwa A., Kaplan W., Koledova E., Savage M.O. (2018). The growth hormone-insulin-like growth factor-I axis in the diagnosis and treatment of growth disorders. Endocr. Connect..

[30] Werner H. (2023). The IGF1 signaling pathway: From basic concepts to therapeutic opportunities. Int. J. Mol. Sci..

[31] Asfour H.A., Allouh M.Z., Said R.S. (2018). Myogenic regulatory factors: The orchestrators of myogenesis after 30 years of discovery. Exp. Biol. Med..

[32] Hernández-Hernández J.M., García-González E.G., Brun C.E., Rudnicki M.A. (2017). The myogenic regulatory factors, determinants of muscle development, cell identity and regeneration. Semin. Cell Dev. Biol..

[33] Fajardo C., De Donato M., Macedo M., Charoonnart P., Saksmerprome V., Yang L., Purton S., Mancera J.M., Costas B. (2024). RNA interference applied to crustacean aquaculture. Biomolecules.

[34] Hannon G.J. (2002). RNA interference. Nature.

[35] Huvenne H., Smagghe G. (2010). Mechanisms of dsRNA uptake in insects and potential of RNAi for pest control: A review. J. Insect Physiol..

[36] Zhu K.Y., Palli S.R. (2020). Mechanisms, applications, and challenges of insect RNA interference. Annu. Rev. Entomol..

[37] Jiang F.W., Fu H.T., Qiao H., Zhang W.Y., Jiang S.F., Xiong Y.W., Sun S.M., Gong Y.S., Jin S.B. (2014). The RNA interference regularity of transformer-2 gene of oriental river prawn *Macrobrachium nipponense*. Chin. Agric. Sci. Bull..

[38] Cui W., Liu X., Zhang S., Li X., Wang Z., Li Y., Zhang M., Wang L., Yu M., Qiao Z. (2025). Identification and ovarian developmental regulation of ribosomal protein S6 kinase in *Macrobrachium nipponense*. Int. J. Biol. Macromol..

[39] Jiang H., Liu X., Li Y., Zhang R., Liu H., Ma X., Wu L., Qiao Z., Li X. (2022). Identification of ribosomal protein L24 (RPL24) from the oriental river prawn, *Macrobrachium nipponense*, and its roles in ovarian development. Comp. Biochem. Physiol. Part A Mol. Integr. Physiol..

[40] Li F.J., Zhang S.Y., Fu C.P., Wang A.L., Zhang D. (2019). Comparative transcriptional analysis and RNA interference reveal immunoregulatory pathways involved in growth of the oriental river prawn *Macrobrachium nipponense*. Comp. Biochem. Physiol. Part D Genom. Proteom..

[41] Sun B., Luo H., Zhao S., Yu J.L., Lv X.T., Yi C., Wang H. (2019). Characterization and expression analysis of a gC1qR gene from *Macrobrachium nipponense* under ammonia-N stress. Aquaculture.

[42] Li M.Y., Qin N., Yuan B.W., Guo M.H., Yang L.K., Tang T., Li F.C., Liu F.S. (2025). Involvement of nerve cord-expressed SVWC2 in pathogen recognition and defense in *Macrobrachium nipponense*. Fish Shellfish Immun..

[43] Ren Q., Huang Y., Liu B.X. (2025). Function of two newly identified Spätzle genes in innate immune response of *Macrobrachium nipponense* against WSSV infection. Fish Shellfish Immun..

[44] Rombel I.T., Sykes K.F., Rayner S., Johnston S.A. (2002). ORF-FINDER: A vector for high-throughput gene identification. Gene.

[45] Boeckmann B., Bairoch A., Apweiler R., Blatter M.C., Estreicher A., Gasteiger E., Martin M.J., Michoud K., O’Donovan C., Phan I. (2003). The SWISS-PROT protein knowledgebase and its supplement TrEMBL in 2003. Nucleic Acids Res..

[46] Marchler-Bauer A., Derbyshire M.K., Gonzales N.R., Lu S., Farideh C., Geer L.Y., Geer R.C., He J., Marc G., Hurwitz D.I. (2015). CDD: NCBI’s conserved domain database. Nucleic Acids Res..

[47] Benson D.A., Cavanaugh M., Clark K., Lipman D.J., Ostell J., Sayers E.W. (2012). GenBank. Nucleic Acids Res..

[48] Thompson J.D., Gibson T.J., Higgins D.G. (2003). Multiple sequence alignment using ClustalW and ClustalX. Curr. Protoc. Bioinform..

[49] Kumar S., Tamura K., Nei M. (1994). MEGA: Molecular evolutionary genetics analysis software for microcomputers. Comput. Appl. Biosci..

[50] Jin S.B., Jiang S.F., Xiong Y.W., Qiao H., Sun S.M., Zhang W.Y., Li F.J., Gong Y.S., Fu H.T. (2014). Molecular cloning of two tropomyosin family genes and expression analysis during development in oriental river prawn, *Macrobrachium nipponense*. Gene.

[51] Hu Y.N., Fu H.T., Qiao H., Sun S.M., Zhang W.Y., Jin S.B., Jiang S.F., Gong Y.S., Xiong Y.W., Wu Y. (2018). Validation and evaluation of reference genes for quantitative real-time PCR in *Macrobrachium nipponense*. Int. J. Mol. Sci..

[52] Livak K.J., Schmittgen T.D. (2001). Analysis of relative gene expression data using real-time quantitative PCR and the 2^−∆∆Ct^ method. Methods.

[53] Zhang S.B., Jiang P., Wang Z.Q., Long S.R., Liu R.D., Zhang X., Yang W., Ren H.J., Cui J. (2016). Dsrna-mediated silencing of nudix hydrolase in *Trichinella spiralis* inhibits the larval invasion and survival in mice. Exp. Parasitol..

